# Supplementary material for: Health services for aboriginal and Torres Strait Islander children in remote Australia: A scoping review
Source: PLOS Glob Public Health. 2023 Feb 13;3(2):e0001140. doi: 10.1371/journal.pgph.0001140 (PMC10022200; doi:10.1371/journal.pgph.0001140)
Supplement: S1 Table — (DOCX) [file pgph.0001140.s001.docx]

**APPENDIX**

**Table 1a: Peer-reviewed literature**

| **Author, Date, Title** | **Location, Setting** | **Objective(s), Study type** | **Key findings** | **Analysis/Gaps** |
| --- | --- | --- | --- | --- |
| Achat, 2010 [1]  General health care service utilisation: Where, when and by whom in a socioeconomically disadvantaged population | Disadvantaged population, heath care use. | Examine the utilisation of and preferences related to health care services by residents of a disadvantaged area and to identify factors associated with levels of current and future use.  Survey. | Disadvantaged communities make only moderate use of specialists and emergency departments, and little routine use of other primary health services. | Effect of length of visit and level of engagement not included in dataset. |
| Adams, 2011 [2]  Starting a perinatal and infant mental health service at Winnunga Nimmityjah | Canberra, Australia, mental health service. | Describe how the contexts of social and emotional wellbeing in Aboriginal and Torres Strait Islander communities impacts on perinatal and infant mental health.  Description of a health service. | The process of starting the clinic and some clinical themes are described. | Shows the need for perinatal and infant mental health services at AHC. |
| Allen, 1996 [3]  Anthill and other injuries: a case for mobile allied health teams to remote Australia | Far North Queensland, Australia, mobile allied health teams | Assess a mobile allied health service visiting the remote western shires of the Peninsula and Torres Strait health region.  Description of a health service. | There is demand for allied health services; mobile remote outreach services are cost effective. | Multidisciplinary outreach teams are cost-effective. |
| Ansari, 2006 [4]  Access to health care and hospitalization for ambulatory care sensitive conditions | Victoria, Australia | Examine the external validity of the indicator Hospitalization for Ambulatory Care Sensitive Conditions (ACSH).  Validation of tool. | Hospitalization for ACSH is an accepted indicator of access to health care and avoidable morbidity. Results support the ACSH indicator. When rural residence was considered, the covariate measuring access was not significant. However, rural residence also may contribute importantly to access. Results suggest both the complexity of the meaning of access and the desirability of further research to validate the ACSH indicator. | The use of ACSH as an indicator of health services access and avoidable morbidity |
| Bailie, 2003 [5]  Preventive medical care in remote Aboriginal communities in the Northern Territory: a follow-up study of the impact of clinical guidelines, computerised recall and reminder systems, and audit and feedback | Northern Territory, Remote Aboriginal Communities | Describe the impact on trends in delivery of preventive medical services following a multifaceted intervention.  Post-intervention evaluation. | Clinical guidelines, computerised recall and reminder systems, and audit and feedback improved service outcomes. | Initial improvements were “not fully sustained over the three-year period” showing that the solution is not simply providing but maintaining an evidence-base service. |
| Bailie, 2008 [6]  Delivery of child health services in Indigenous communities: implications for the federal government's emergency intervention in the Northern Territory | NT, Australia, Aboriginal communities | Describe delivery of child health services in Australian Aboriginal communities, identifies gaps in services required to improve the health of Aboriginal children.  Cross-sectional baseline audit. | Ineffective systems for follow-up mean that screening children for disease and adverse social circumstances will result in little or no benefit. | Shows existing services are not enough and that there is a great need for long term, sustainable services |
| Bar-Zeev, 2012 [7]  Use of health services by remote dwelling Aboriginal infants in tropical northern Australia: a retrospective cohort study | NT, Australia, Aboriginal communities | Describe health service utilisation patterns at the primary and referral level by remote dwelling Aboriginal infants from northern Australia.  Retrospective cohort study. | Hospitalisation rate is high and admissions commence early in life, visits to the remote primary health centre are frequent. Half of all presentations are for new problems. |  |
| Barclay, 2014 [8]  Improving Aboriginal maternal and infant health services in the 'Top End' of Australia; synthesis of the findings of a health services research program aimed at engaging stakeholders, developing research capacity and embedding change | Northern Territory, Australia, remote maternity services | Present a five-year collaborative program between stakeholders and researchers that led to sustainable improvements in the maternity services for remote-dwelling Aboriginal women and their infants in the Top End (TE) of Australia.  Service audit. | System-wide problems were identified, causing poor quality of infant services: unacceptable standards of infant care and parent support; no relationship between volume and acuity of presentations and staff numbers or required skills for providing care for infants; and an ‘outpatient’ model of care.  Insufficient/absent Aboriginal leadership and inadequate coordination between remote and tertiary services. | Strength: worked closely with NT Health throughout: NT Health requested some parts of the study and so were invested in findings. |
| Barr, 2018 [9]  A systematic review of services to DHH children in rural and remote regions | Australia-wide, services for hearing impaired | Document service provision to children with hearing loss in regional, rural and remote Australia.  Systematic review. | Reduced quality and frequency of service in rural and remote areas. | Small number of relevant studies. |
| Breen, 2018 [10]  Significant reductions in tertiary hospital encounters and less travel for families after implementation of Paediatric Care Coordination in Australia | Australia-wide, care coordination | Evaluate the impacts of Care Coordination on tertiary hospital service use and family outcomes.  Cohort study. | The Care Coordination service has clear benefits for the tertiary paediatric hospital network and for families. Au$4.9 million was saved over 2 years due to prevented hospital encounters. | Took place in very well-funded tertiary network so less relevant for rural/remote; however rural/remote often travel to tertiary centres and this program shows reduction in this travel burden. |
| Buckley, 2012 [11]  Videoconferencing could reduce the number of mental health patients transferred from outlying facilities to a regional mental health unit | Regional NSW, acute mental health videoconferencing | Determine if adding video link to the existing phone connection would decrease transfer to the central mental health unit.  Cohort study. | The ability for the psychiatrist or senior mental health clinician to see mental health patients via videoconferencing was associated with a reduced probability of the patient being transferred. This satisfies the preference of patients to remain in their community and access mental health services. | Videoconferencing allows patients to remain in their communities whilst accessing mental health services and reduces the rate of transferrals |
| Chandradasa, 2019 [12]  Collaborative networking between regional child mental health, paediatric and educational services in Gippsland, Australia: An online survey | Reginal Victoria, Australia, survey | Survey of health professionals on utility of collaborative networking between regional child mental health, paediatric and educational services in Gippsland, Australia.  Survey. | Survey found that monthly meetings between CAMHS, Department of Education, paediatricians was beneficial. | Survey of existing regular inter-disciplinary meeting; positive review may be expected from those already organising this meeting.  Patient/family survey to determine what matters for them would strengthen study. |
| Cord-Udy, 2004 [13]  The Medical Specialist Outreach Assistance Programme in South Australia at 2 years | Remote SA, Australia, psychiatry outreach | Describes the progress and achievements of the psychiatric services developed by the Medical Specialist Outreach Assistance Programme in South Australia over the last 2 years.  Service audit. | During this period there has been an expansion of visiting psychiatric services to South Australia’s rural and remote communities. There is increased access to Child and Indigenous services. | Psych services – the benefits of specialist outreach |
| Couzos, 2004 [14]  Practical measures that improve human rights - towards health equity for Aboriginal children | Australia-wide | Detail the requirements, in terms of health policy frameworks and preventative programs, for Australian Aboriginal maternal and child health.  Commentary. | Recommendations include the need for the adoption of a policy framework for maternal and child health, concomitant national performance indicators, nutritional supplementation programs, multifaceted measures to enhance the immunisation coverage of Aboriginal children and preventive child health assessments. | Improve health policy and programs; improve the AHS and the child and mental health framework and KPIs |
| D'Aprano, 2016 [15]  Challenges in monitoring the development of young children in remote Aboriginal health services: clinical audit findings and recommendations for improving practice | NT, Australia, remote Aboriginal Health Centres | Describe developmental monitoring practice in two remote Australian Aboriginal primary healthcare services and identify gaps in the delivery of developmental monitoring services.  Service audit. | Developmental checks were more likely among children who attended services more regularly.  Inconsistent documentation.  Need for a systems-wide approach to the delivery and recording of developmental monitoring services. This will require routine training of remote Aboriginal health workers and remote area nurses in developmental monitoring practice including the use of a culturally appropriate, structured developmental screening measure. | Retrospective audit with difficulty differentiating inadequate care from poor documentation; authors note that poor documentation will in itself impact continuity of care. |
| Dossetor, 1999 [16]  A child and adolescent psychiatric outreach service for rural New South Wales: a telemedicine pilot study | Rural NSW, Australia, psychiatry telehealth outreach | Examines the feasibility of a tertiary outreach service in child and adolescent psychiatry to two rural health centres in New South Wales, Australia.  Prospective cohort study. | Telepsychiatry provides access to a flexible, effective tertiary service for those with special, complex needs, including the disadvantaged or isolated. It makes a valuable, economic contribution to supporting and educating rural health professionals, thereby enriching rural mental health services. |  |
| Dossetor, 2017 [17]  Paediatric hospital admissions in Indigenous children: a population-based study in remote Australia | Kimberley Region, WA, Australia, rural hospital | Analysis of hospital admissions of a predominantly Aboriginal cohort of children in the remote Fitzroy Valley in Western Australia during the first 7 years of life.  Descriptive, quantitative. | In the Fitzroy Valley 70% of children were hospitalised at least once before age 7 years and over one third of admissions were in infants. Infections were the most common reason for admission in all age groups but comorbidities were common  Many hospitalizations were feasibly preventable. | Misses children transferred directly to larger hospitals. |
| Dossetor, 2019 [18]  Review of Aboriginal child health services in remote Western Australia identifies challenges and informs solutions | Kimberley region, Australia, health services | Identify and map child health services in the very remote Fitzroy Valley, West Kimberley, and document barriers to effective service delivery.  Service audit. | There is no document providing a comprehensive overview of child health services in Fitzroy Valley.  The need to attend to acute illness acts to deprioritise crucial primary and preventative health care.  Critical shortage of Aboriginal Health Workers. | Strength: produced in collaboration with local community. |
| Dossetor, 2021 [19]  Emergency department use in a rural Australian setting: are the factors prompting attendance appropriate? | Australia, a rural hospital | Examine if the reasons for attendance at rural and remote ED are appropriate.  Survey. | The results highlighted the importance of the rural hospital ED as an additional and alternate service to existing primary care, particularly outside business hours. ED was utilised for clinically non-emergent conditions, which was justified given availability to services. | Highlights the importance of services additional to PHC services in remote areas. |
| Edmond, 2018 [20]  Quality of social and emotional wellbeing services for families of young Indigenous children attending primary care centers; a cross sectional analysis | Australia-wide, PHC | Assess delivery of social and emotional wellbeing services to the families of young (3-11 months) and older (12-59 months) Indigenous children attending primary care centres.  Cross-sectional study. | The families of young Indigenous children appear to receive priority for social and emotional wellbeing care in Australian primary care centres, however many Indigenous families are not receiving services. | Auditors were clinic staff with standardised training however may be increased inter-rater variability. Participation was voluntary à possible selection bias. |
| Edmond, 2019 [21]  Improving developmental care in primary practice for disadvantaged children | Australia-wide, PHC | Assess if sustained participation in continuous quality improvement (CQI) activities could improve delivery of 'basic developmental care' to disadvantaged children in primary care settings.  Cross-sectional study. | Sustained CQI can improve basic developmental care in primary care settings. However, many disadvantaged children are not receiving services. | No interviews with patients/families; missed patients who are not presenting for care at all. |
| Flabouris, 2012 [22]  Accessibility of the Australian population to an ICU, and of ICUs to each other | Australia-wide, ICUs | Uses a geographic information system to illustrate the geospatial relationship of the Australian population to intensive care resources.  Spatial survey. | The distribution of Australian ICUs and the Australian population was similar. However, accessibility varied by state/territory. |  |
| Francis, 2016 [23]  The impact of socioeconomic status and geographic remoteness on access to pre-emptive kidney transplantation and transplant outcomes among children | Australia-wide, nephrology | Determine whether access to pre-emptive transplantation and transplant outcomes differ according to SES and geographic remoteness in Australia.  Cohort study. | In Australia, children from regional or remote regions are much less likely to receive pre-emptive kidney transplantation.  Strategies such as improved access to nephrology services through expanding the scope of outreach clinics, and support for regional paediatricians to promote early referral may ameliorate this inequity. | Subgroup analyses may be underpowered; used a robust database. |
| Garne, 2009 [24]  Frequent users of the Royal Flying Doctor Service primary clinic and aeromedical services in remote New South Wales: a quality study | Rural/remote NSW, Australia, retrieval/outreach | Examine activity patterns of the Royal Flying Doctor Service of Australia (RFDS) in far western New South Wales and to determine whether frequent use of RFDS services, particularly emergency evacuations, is a useful indicator of patients who may benefit from care planning and review.  Audit. | Conclusions: Simple, practical clinical review systems can help health care organisations in rural and remote communities to achieve better outcomes by identifying patients who may benefit from planned care. |  |
| Geelhoed, 2008 [25]  Positive impact of increased number of emergency consultants | Australia, Paediatric emergency department | Determine the impact of increased paediatric emergency deparment consultants.  Cohort study. | The provision of additional consultant medical staff in a paediatric ED coincided with a decrease in the percentage of children admitted, complaints to the department and average waiting times, and was cost effective. | Increasing use of ED services decreased the proportion of kids admitted to hospital and waiting times and was cost effective. |
| Gruen, 2006 [26]  Specialist outreach to isolated and disadvantaged communities: a population-based study | Northern Australia, 3 remote Indigenous communities | Assess the effects of outreach clinics on access, referral patterns, and care outcomes in remote communities in Australia.  Cohort study. | Specialist outreach visits to remote Indigenous communities in Australia improve access to specialist consultations and procedures without increasing elective referrals or demands for hospital inpatient services. | Not paediatric-focused |
| Gunasekera, 2009 [27]  Otitis media in Aboriginal children: the discordance between burden of illness and access to services in rural/remote and urban Australia | All Australian Aboriginal medical services | Compare burden of otitis media (OM) managed by Aboriginal Medical Service (AMS) practitioners and the availability of specialist ear health services in rural/remote versus urban Australian settings.  Survey of AMS practitioners/services. | Rural/Remote AMS practitioners manage a greater OM burden than urban AMS practitioners. One in five rural/remote Aboriginal children wait longer than recommended for audiology testing. | Shortage of health services for the burden of disease. |
| Hayman, 2010 [28]  Strategies to improve indigenous access for urban and regional populations to health services | Queensland, PHC | Describe strategies to improve indigenous health services access.  Commentary. | Community consultation and participation were the main ingredients to improving indigenous access to the service. All primary health care services working in Aboriginal and Torres Strait Islander health settings should have access to funded continuous quality improvement activities. | Not solely paediatric-focused. New data. |
| Henderson, 2008 [29]  Review of community paediatrics, the Central Australian Remote Health Service, Alice Springs | Central Australia, outreach paediatrics | Review outreach community paediatric service operating out of Central Australian Outreach Remote Health Service.  Service audit. | Wide-ranging findings and recommendations relating to relationship of hospital and community-based paediatric services. |  |
| Humphreys, 2009 [30]  Key considerations in delivering appropriate and accessible health care for rural and remote populations: discussant overview | Australia-wide | Provide an overview of papers discussing optimal service delivery models for rural and remote Australia.  Narrative discussion. | Systemic change is required to ensure equitable access to health care services in small rural and remote communities. | Requirements for equitable access to health care should be based on:   - overarching health goals - agreed service requirements - recognition of how rural and remote health contexts impact upon health service provision - the constraints limiting health service responses |
| Hussain, 2015 [31]  Parental perceptions of information needs and service provision for children with developmental disabilities in rural Australia | Australia-wide, consumer advocacy | Give voice to parents regarding challenges caring for children with developmental disabilities, faced by them due to their location in rural regions.  Survey, interviews. | Feasible options within resource constraints to improve support include focused in-service training to narrow the information gap, improve provider-client interaction around attitudinal issues, and uptake of tele-health services.  Setting up of parent-professional support groups as well peer support groups using digital technologies will help reduce the sense of isolation for rural carers and minimise impediments related to travelling long distances. | Interviews had no indigenous participants. |
| Jeffery, 2011 [32]  Responding to rural health needs through community participation: addressing the concerns of children and young adults | Rural Vic, Australia, health service | Detail a needs analysis of a small rural health service in 2008 to identify gaps in service delivery and duplication of services. This exercise was intended to inform strategic direction but the result was consumer and community consultation and outcomes that far exceeded everyone’s expectations.  Participatory Rural Appraisal. | The project described resulted in targeted, purposeful action regarding community engagement, and the findings and outcomes are reflective of this. | Partnerships between HS and communities |
| Johns, 2010 [33]  Early childhood service development and intersectoral collaboration in rural Australia | Tasmania, Australia, in three small communities | Examine community-based intersectoral collaborations involving government and non-government organisations from the health and allied health, education and community service sectors.  Qualitative study. | Three groups of factors influence collaborations: social capital, leadership and environmental. Internal and external leaders are needed. | Infant service development and function depends on: Effective models of early childhood development require strong local and external leadership; harness the skills, knowledge and resources of internal and external leaders; the ability to this empowers them to take control/ownership of health & wellbeing |
| Jones, 2018 [34]  Rural and remote speech-language pathology service inequities: An Australian human rights dilemma | Australia-wide, review | Examine lack of health services, specifically speech-language pathology with a human rights approach. Literature review.  Narrative discussion. | NA | Narrative synthesis of literature |
| Josif, 2017 [35]  The quality of health services provided to remote dwelling aboriginal infants in the top end of northern Australia following health system changes: A qualitative analysis | Top End, Australia, Aboriginal communities | Investigate service quality, from the clinicians’ perspective and as observed and recorded by the researcher, in two large Aboriginal communities in the Top End of northern Australia following health system changes.  Observational study. | A range of negative factors persisted beyond the examined health system change: ineffective service delivery, inadequate staffing, culturally unsafe practices.  The six themes identified in the data: ‘very adhoc’, ‘swallowed by acute’, ‘going under’, ‘a flux’, ‘a huge barrier’ and ‘them and us’ illustrate how these factors continue, and when combined portray a ‘very chaotic system’. | A few hours of observation in two communities brings into question the saturation and generalisability of identified themes. |
| Kang, 2020 [36]  The relationship between having a regular general practitioner (GP) and the experience of healthcare barriers: a cross-sectional study among young people in NSW, Australia, with oversampling from marginalised groups | NSW, Australia, PHC | Explore the association between having a regular GP and experience of healthcare barriers and attitudes to health system navigation among young people in New South Wales (NSW), Australia.  ‘Over-sampling’ of ATSI and rural/remote groups.  Cross-sectional study. | Having a regular GP was associated with having more positive attitudes to health system navigation.  Those with regular GP were less likely impacted by cost, other structural barriers, feeling judged, and not knowing which service to go to. | Oversampled ATSI and rural/remote; Cross-sectional design though strengthened by qualitative component. |
| Langbecker, 2019 [37]  Impact of school-based allied health therapy via telehealth on children's speech and language, class participation and educational outcomes | QLD, Australia, schools | Examine the impact of school-based allied health therapy via telehealth on children's speech and language, class participation and educational outcomes.  Observational study. | Delivering allied health therapy by videoconference may enable children to better engage with schooling. Differences in improvements by grade suggest improvements may be maximised by targeting therapy at certain year levels. This model may be useful in areas with limited access to allied health services. | Didn’t compare with telehealth with standard in-person service. Didn’t note if participants were ATSI, or how program could work for ATSI students |
| Larson, 2010 [38]  Aboriginal maternal and child project: strengths and needs analysis | WA, Australia, Epidemiological | Strengths and needs analysis of aboriginal maternal and child health.  Strengths and Needs analysis. | Aboriginal community-controlled health services provide a value maternal health service and have the potential to contribute to effective child  health care. Greater investment in the basic infrastructure necessary to deliver services – the space,  funding and partnerships with other health services – is directly translated into improved quality of  service provision. | Recommendations for models of care, communication & infrastructure, access, workforce, integration, and referral for ATSI mothers and babies. |
| Lenthall S, 2011 [39] Nursing workforce in very remote Australia, characteristics and key issues | Remote Australia, nursing workforce | Describe the nursing workforce in very remote Australia, characteristics, and key issues.  Descriptive and narrative discussion. | The workforce is ageing, the numbers of nurses per population has fallen and the numbers of midwives and child health nurses have dropped significantly over the last 15 years. As many of these nurses work in Indigenous communities, if these trends continue it is likely to have a negative effect on ‘closing the gap’ in Indigenous health outcomes. | Decrease in nursing workforce particularly remote, paediatric nurses |
| Margolis, 2012 [40]  Is Fly in/Fly out (FIFO) a viable interim solution to address remote medical workforce shortages? | Remote Australia | Describe Fly In Fly Out (FIFO) viability, anecdotal synthesis.  Narrative discussion. | Effective FIFO needs commitment from the sponsoring organisation for short, balanced, flexible, family friendly rosters. A positive organizational structure with effective communication between management and front-line staff is also required. FIFO can capitalise on mid-career doctors who are experienced in remote work but have moved to cities. | Advocates for the FIFO model – to address rural health service workforce shortages. |
| McCalman, 2012 [41]  Applying what works: a systematic search of the transfer and implementation of promising Indigenous Australian health services and programs | Australia-wide | Evaluate transfer and implementation of Indigenous health services.  Systematic review. | Few studies focus on the process of transfer and implementation or the effectiveness of the service or program in the new setting. | Once implemented, services aren’t being assessed on their effectiveness. This requires active partnerships between services and researchers. |
| McKeown, 2011 [42]  Evaluating aboriginal primary health care services using national key performance indicators: What has happened to the social theories of aboriginal health? | Australia-wide, primary health care (PHC) | Examine whether KPIs focused on process and outcome of clinical service delivery are appropriate to Aboriginal-controlled PHCs?  Commentary. | Evaluation frameworks for Aboriginal PHC services need to encompass wider PHC frameworks rather than a narrower biomedical approach to improving health. | Suggests that using the KPI to evaluate Aboriginal PHC services won’t provide an accurate reflection of their contribution to improving health. |
| Medlin, 2014 [43]  Indigenous respiratory outreach care (IROC): Enhancing respiratory health in rural and remote aboriginal and Torres strait islander communities | QLD, Australia, specialist outreach | Assess the Indigenous Respiratory Outreach (nurse-led) Care (IROC) (adults and paediatrics) in rural/remote ATSI. Program evaluation  Cohort study. | IROC started in 2011, now 12 community sites. Early data shows communities like it and it has picked up previously undetected paediatric chronic lung disease. IROC provides a successful model for achieving this goal that is readily transferrable to other regions in Australia. | Preliminary data |
| Mitchell, 2018 [44]  Transition to adult care for Aboriginal children with rheumatic fever: a review informed by a focussed ethnography in northern Australia | Northern Territory, Australia, remote communities | Describe transition to adult care for children with chronic conditions and consider applicability to the care of children.  Narrative review, ethnographic study of four remote communities. | Recommended tailoring of transition care to engage and value local navigators who can address language and cultural barriers to provide a sustainable alternative to transition coordinators in mainstream programs. This has potential to improve care without further burdening overstretched clinical resources. | Explicitly took account of power imbalances. Assessed an evidenced program in new setting |
| Mitchinson, 2019 [45]  Anaemia in disadvantaged children aged under five years; quality of care in primary practice | Australia-wide, PHC | Assess quality of care of anaemic disadvantaged children.  Cross-sectional study. | The burden of anaemia and quality of care for disadvantaged Indigenous children was concerning across all remote and urban locations assessed in this study. | Self-selected study group may limit generalisability; Only 70% children screened for anaemia, remainder may be more severe (differential error) |
| Moffatt, 2010 [46]  The reported benefits of telehealth for rural Australians | Australia-wide | Identify reported benefits attributed to telehealth for people living and professionals working in rural and remote areas of Australia.  Narrative review. | Telehealth may decrease urban–rural health disparities and improve workforce retention. Benefits of telehealth for patients: cheaper, convenient, better access, better quality; for clinicians: educational opportunities, experimental learning, networking, collaboration. |  |
| Morgan, 2012 [47]  QT babies: Neonatal care in the Queensland tropics | Far North QLD, Australia | Review the requirements of this diverse and geographically isolated population and how they are met by the team at The Townsville Hospital.  Narrative discussion. | The challenge of providing neonatal care to this unique population continues to be successfully achieved by the dedicated extended multidisciplinary team at The Townsville Hospital. | Neonatal care is successfully achieved at the Townsville hospital ATSI, shows the success of a multidisciplinary team |
| Nancarrow, 2015 [48]  Models of care involving district hospitals: a rapid review to inform the Australian rural and remote context | Australia-wide | Identify models of care that incorporate district hospitals and have relevance to the Australian rural and remote context.  Service audit. | District hospitals are important symbolically and functionally for small towns. They assist in vertical integration of health services. | Study commissioned for policy development. Noted that literature focused on rural health systems more broadly, not the target of review: ‘district hospitals’ |
| Nguyen, 2015 [49]  Cost-effectiveness analysis of a mobile ear screening and surveillance service versus an outreach screening, surveillance and surgical service for Indigenous children in Australia | Rural Queensland, Australia, ENT mobile outreach | Evaluate the cost-effectiveness of the community-based mobile telemedicine-enabled screening and surveillance (MTESS) service using a lifetime Markov model that compares two options: (i) the Deadly Ears Program alone (current practice involving an outreach ENT surgical service and screening program), and (ii) the Deadly Ears Program supplemented with the MTESS service.  Economic analysis. | The MTESS service is cost-effective. It presents an opportunity to resolve major issues confronting Australia’s health system such as the inequitable provision and access to quality healthcare for rural and remotes communities, and for Indigenous Australians. | Modelling relied on nascent epidemiology of ear disease in Indigenous People. |
| Nguyen, 2018 [50]  Feasibility of Implementing Infant Home Visiting in a Central Australian Aboriginal Community | Central Australia, home visits | Assess the feasibility of Implementing Infant Home Visiting in a Central Australian Aboriginal Community (as is/was done in Alice Springs since 2009).  Descriptive, cohort study. | This study finds it could be done; needs outcome evaluation to test hypothesised benefits. |  |
| O'Callaghan, 2005 [51]  Consumers' proposed solutions to barriers to access of rural and remote speech pathology services | Remote NSW, Australia, paediatrics speech pathology | Investigate solutions to perceived barriers of consumers when accessing paediatric speech pathology services in rural and remote New South Wales (NSW).  Survey design. | Consumer-based solutions to barriers to access to maximize the effectiveness of sparse rural and remote speech pathology services, by matching the beliefs and expectations of consumers with the characteristics of services provided. | Barriers to services – allied health, speech paths. |
| O’Kane, 2004 [52]  Towards a needs based mental health resource allocation and service development in rural and remote Australia | Central Australia, Remote mental health services | Develop a transparent, needs- and evidence-based framework to guide resource allocation and mental health service development in rural and remote settings.  Review of services. | Existing funding allocation was half that required for adequate, equitable level of care. | Demonstrates great need for mental services, particularly for children, adolescents and remote-dwelling Aboriginal people. |
| Ou, 2010 [53]  The comparison of health status and health services utilisation between Indigenous and non-Indigenous infants in Australia | Australia-wide, LSAC analysis | Describe differences in health services utilisation and the associated risk factors between Indigenous and non-Indigenous infants at a national level in Australia.  Analysis of a national longitudinal dataset. | Indigenous infants use less healthcare than non-Indgienous. Determining factors include mothers’ characteristics and socio-economic status. | ATSI children are more likely to use a hospital outpatient clinic than: maternal & child health centre, help lines, maternal and child health nurse visits, GP, or paediatrician |
| Peiris, 2006 [54]  Aeromedical evacuations from an east Arnhem Land community 2003-2005: The impact on a primary health care centre | Arnhem Land, Australia, PHC evacuations | Describe the profile and impact of aeromedical evacuations by Royal Flying Doctor Service in remote Indigenous communities.  Service audit. | Aeromedical evacuations burden primary health centres. Meeting this burden competes with regular clinic duties. Clinic staff are regularly required to provide hospital-level acute care, often for several hours at a time. | High service usage but is expensive and could be decreased by improving availability of remote area health centres. |
| Phillips, 2014 [55]  Can mobile phone multimedia  messages and text messages improve clinic attendance for Aboriginal children with chronic otitis media? A randomised controlled trial | Remote NT, Australia, community | Examine effect of phone multimedia messages (MMS) to families of Indigenous children with tympanic membrane perforation (TMP): (i) increase clinic attendance; (ii) improve ear health; and (iii) provide a culturally appropriate method of health promotion?’  Randomised-controlled trial. | No improvement in clinic attendance or ear health. But this study shows that MMS in local languages can be culturally appropriate. | Affected by social conditions: didn’t own, or couldn’t regularly access mobile phone |
| Ruben, 1998 [56]  The case mix system of hospital funding can further disadvantage Aboriginal children | NT, Australia, funding model. | Examine effect of mixed hospital funding using national averages and national cost weights as benchmarks for length of stay and funding.  Retrospective cohort study. | ATSI children had more comorbidities, and prolonged hospital stays. These results confirm clinical impressions about disease patterns and length of hospital stay in ATSI children, and highlight the problems of imposing a case mix classification system for a "typical" Australian population on a region with a high proportion of people of ATSI descent. | Explores problems associated with case mix system of hospital funding for complex patients. |
| Schofield, 2009 [57]  Multidisciplinary management of complex care | Northern New South Wales, Australia, in community | Describe the advantages of multidisciplinary care in terms of patient outcomes, clinician satisfaction and system efficiency are considered with reference to an innovative rural multidisciplinary model that highlights how these positive outcomes can be achieved.  Pre and post descriptive study. | Multidisciplinary care can provide better health outcomes and a more satisfying work environment for health professionals. Especially in rural areas where the only colleagues may be from other disciplines. |  |
| Seidler, 2020 [58]  Patterns of Youth Mental Health Service Use and Discontinuation: Population Data from Australia’s Headspace Model of Care | Australia-wide, mental health services. | Explore sociodemographic predictors of attendance and discontinuation of mental health services in a large, population-based sample.  Cohort study. | Sociodemographic factors were found to be associated with treatment discontinuation, and some young people followed a pathway in and out of mental health treatment. Further exploration is needed to determine the appropriate length and type of care for specific socio- demographic groups and how best to tailor treatment accordingly. | Used existing dataset (minimum data set; MDS). |
| Smith, 2010 [59]  Clinical services and professional support: a review of mobile telepaediatric services in Queensland | Rural and remote QLD, Australia, health services | Review of telepaediatric service records to determine which clinical and educational services had been delivered through the mobile videoconference systems.  Narrative review. | Our service model provided a streamlined method of delivering specialist health services to children and families living in rural and remote regions of Queensland. |  |
| Thomas, 2014 [60]  What core primary health care services should be available to Australians living in rural and remote communities? | Australia-wide | Describe what core primary health care services should be available to rural/remote Australians.  Delphi survey of remote health experts including policy-makers, academics, clinicians and consumers. | This study identifies a basket of PHC services that consumers in rural and remote communities can expect to access. It provides rigorously derived evidence that will contribute to a more systematic approach to PHC service planning and availability and will assist policy makers in the allocation of scarce resources necessary to improve the health outcomes of residents of rural and remote areas. | Only 4 (10%) Delphi panellists were from community (‘consumer representatives’). |
| Veit, 1996 [61]  Barriers to effective primary health care for adolescents | Victorian GPs | Describe general practitioners' (GPs) perceptions of barriers in the health care system that hinder provision of effective adolescent health care; and assess the training needs of GPs for a future education program in adolescent health.  Survey of GPs. | Medicare cards should be automatically issued to adolescents from age 16 years to improve their access to health care. Revision of Medicare rebate system and clarification of HIC's investigative functions may improve GP's effectiveness. |  |
| Verdon, 2011 [62]  An investigation of equity of rural speech-language pathology services for children: a geographic perspective | NSW and Victoria, Australia, speech pathologists | Assess speech pathology services in rural areas of New South Wales and Victoria/  Structured interviews with 74 speech- language pathologists. | Almost all (98.60%) localities are underserviced. Fifty kilometres is the critical maximum distance past which consumers become unable or unwilling to travel to access weekly rural speech-language pathology services in rural New South Wales and Victoria. | Speech pathology may be not representative of wide access. |
| Wakerman, 2008 [63]  Primary health care delivery models in rural and remote Australia: a systematic review | Australia-wide | Describe what health service models were reported to work, where they worked and why.  Systematic review. | Sustainable models may address diseconomies of scale which result from large distances and small dispersed populations. The authors developed a conceptual framework for development of sustainable remote PHC models. | Systematic review of PHC models in remote Australia enabled development of a conceptual framework to help in the sustainability of these PHC models. |
| Wakerman, 2009 [64]  Innovative rural and remote primary health care models: what do we know and what are the research priorities? | Australia-wide | Examine literature on ‘innovative’ primary health care models in rural and remote areas to identify areas where knowledge is lacking and describes future research priorities.  Narrative synthesis. | Health services evaluation should focus on: optimal financing systems, the optimal range and mix of providers, supports for team practice, appropriate community participation mechanisms, improved health information systems and relevant performance indicators. | Need for evaluation of services to inform best practice. |
| Wakerman, 2009 [65]  Features of effective primary health care models in rural and remote Australia: a case-study analysis | Australia-wide, primary health care | Describe the factors and processes that facilitate or inhibit implementation, sustainability, and generalisation of effective models of primary health care (PHC) service delivery in rural and remote Australia.  Literature review, interviews, direct observation. | In Australia, establishment of a single national health system, operationalised at a regional level, would obviate much of the current inefficiency and poor coordination. | Highlights need for improved:   - governance - management - community involvement |
| Ward, 2006 [66]  Correlations of siblings' and mothers' utilisation of primary and hospital health care: A record linkage study in Western Australia | WA, Australia | Examine the relationships between the utilisation of health care by siblings and mothers over 1984-97.  Observational study. | Strong relationship between siblings’ use of GPs and a weaker but still significant association between the hospital admissions of siblings. Suggests the presence of intergenerational correlation of morbidity; that behavioural factors are not sufficiently explanatory. | Correlation between mothers and children, siblings for GP and Hospital service usage. |
| Warwick, 2019 [67]  Young Aboriginal People’s Perspective on Access to Health Care in Remote Australia: Hearing Their Voices | Kimberley region, WA, Australia, Aboriginal Health Service | Identify barriers and enablers of access for adolescent and young adult Aboriginal people to Derby Aboriginal Health Service (DAHS), a remote Western Australian Aboriginal Community Controlled Health Service (ACCHSs), to assist in improving access.  Qualitative study. | Improving youth engagement seems to be central to increasing acceptability and, hence, use. This requires that staff able to engage with young people are recruited, trained, and retained. | Respondents were patients/consumers. Used local researchers to collect data however researchers were also health service staff which may pressure respondents to reply favourably and introduce bias into results. |
| Whelan, 2013 [68]  Health Services Use and Lifestyle Choices of Indigenous and non-Indigenous Australians | Australia-wide | Analyse national data to examine whether Indigenous Australians make different lifestyle choices and health services use than non-Indigenous Australians.  Observational database study. | Indigenous Australians are more likely to make poorer lifestyle choices but are more likely to use health services. These results are magnified for Indigenous Australians who live in remote areas. | Focuses on adults. |
| Williams, 2004 [69]  Paediatric outreach services | Australia-wide | Describe Paediatric outreach services.  Narrative review. | A combination of telehealth clinics and outreach visits may be most efficient for delivering paediatric specialty and subspecialty care to remote Australia. | Synthesis but nil new data. |
| Zwi, 2011 [70]  Prioritising health services for high risk and disadvantaged children | Australia wide | Document prioritisation strategies for health services for vulnerable children, map current prioritisation methods in an urban area health service, and analyse the views of service directors, clinicians, and intake workers regarding the ethics and practicalities of prioritising care.  Narrative review. | Health staff favour prioritisation strategies for vulnerable children. Prioritisation strategies can burden staff and so require extra resources. |  |

**Table 1b: Source of grey literature**

| **Source** | **Overview** | **Reports used** |
| --- | --- | --- |
| A Country Health Service: Kimberley Health | Western Australia rural health organisation | - Aboriginal Health Profile: Kimberley Health Region, 2012 [71]  - Kimberley Health Profile 2018 [72]  - Child Population Profile, Health and Wellbeing Surveillance System, 2009: Kimberley health region [73] |
| Australian Indigenous Health Infonet | Aims to make knowledge available to health organisations | - Overview of Aboriginal and Torres Strait Islander health status, 2020 [74]  - Summary of Aboriginal and Torres Strait Islander health status 2018 [75] |
| Australian Institute of Health and Welfare | Reports relating to health workforce stats, expenditure, Aboriginal community-controlled health services, ATSI health performance framework reports for each state and territory. Majority national level data analysis, some state level comparative | - Expenditure on health for Aboriginal and Torres Strait Islander people 2008-09: an analysis by remoteness and disease [76]  - Access to health services for Aboriginal and Torres Strait Islander people [77]  - Aboriginal and Torres Strait Islander health services report, 2010-11: OATSIH services reporting [78]  - Healthy for Life—Aboriginal Community Controlled Health Services: report card  [79]  -Aboriginal and Torres Strait Islander Health Performance Framework 2013: Western Australia [80]  - Health workforce 2020 [81] |
| Australian Medical Association | Peak body for medical professionals in Australia | - Ending the Cycle of Vulnerability: The Health of Indigenous Children [82] |
| Australian Primary Health |  | - A systematic review of primary health care delivery models in rural and remote Australia 1993 -2006 [83]  - Interventions to prevent growth faltering in remote Indigenous communities [84] |
| Boab Health | Includes the Kimberley Division of General Practice | - Kimberley Primary Health Care Sustainability Study 2008 – 2030 [85] |
| Centre for Aboriginal Economic Policy Research | Think tank based at Australian National University | - Human capabilities and child development: Some inferences using the 2008 NATSISS [86]  - An ethnography of changes in child rearing over time in the Ngaanyatjarra Lands: Implications for policy development on health in early childhood [87] |
| Commonwealth Department of Families, Housing, Community Services and Indigenous Affairs | National government department | - Indigenous reform 2011-12: comparing performance across Australia [88] |
| Commonwealth Department of Health and Ageing | National government department | - Primary health care in rural and remote Australia: achieving equity of access and outcomes through national reform: A discussion paper [89]  - Evaluation of the Child Health Check Initiative and the Expanding Health Service Delivery Initiative: Summary Report [90] |
| Commonwealth House of Representatives Parliamentary Committees | Indigenous health reported in across several Parliamentary Committee Inquiries | - Health is life: inquiry into Indigenous health [91]  - Inquiry into Health Funding Report: The Blame Game [92] |
| Commonwealth Senate Committee | Senate Select Committee on Regional and Remote Indigenous Communities | - Indigenous Australians, Incarceration and the Criminal Justice System [93] |
| Health Workforce Australia | National research and policy development relating to the development of a sustainable health workforce across Australia | - National Rural and Remote Workforce Innovation and Reform Strategy [94]  - Health Workforce 2025 – Doctors, Nurses and Midwives – Volume 2 [95]  - Growing Our Future: the Aboriginal and Torres Strait Islander Health Worker Project Final Report [96] |
| KAMSC – Kimberley Aboriginal Medical Services Council | Aboriginal controlled coordination group | - Regional Aboriginal Health Plan: Aboriginal Health in the Kimberley: current circumstances and future directions [97]  - Kimberley Aboriginal Primary Health Plan 2012-2015 [98] |
| Lowitja Institute | Aboriginal organisation promoting research and knowledge translation | -Assessing Cost-Effectiveness in Prevention (ACE–Prevention): Final Report. [99]  - Australian Indigenous Health - Within an International Context [100]  - An evaluation of the benefits of swimming pools for the hearing and ear health status of young Indigenous Australians: a whole-of-population study across multiple remote Indigenous communities [101]  - Leadership and legacy through crises: keeping our mob safe Close the Gap campaign report 2021 [102] |
| Menzies School of Health Research | Research organisation in the Northern Territory | - Effective Integration of Services for Children and Families: Making it Happen [103] |
| National Rural Health Conference 2009 | Run through the National Rural Health Alliance | - Pitstop: one stop community health shop for Indigenous families in the West Pilbara [104] |
| Northern Territory Department of Health and Community Services Digital library | Many policy documents relating to child health, and material for health staff to draw on | - Child Malnutrition / Failure to Thrive Project 2008: Final Report [105] |
| Office for Aboriginal and Torres Strait Islander Health | Produces information on  performance of States and Territories vis-à-vis Closing the Gap targets,  Clinical guidelines, and  Health programs | - Improving Health in Aboriginal and Torres Strait Islander Mothers, Babies and Young Children - A Literature Review [106]  - Evaluation of the Child Health Check Initiative and the Expanding Health Service Delivery Initiative: Summary Report [90]  - The link between primary health care and health outcomes for Aboriginal and Torres Strait Islander Australians [107]  - Better Health Care: Studies in the successful delivery of primary health care services for Aboriginal and Torres Strait Islander Australians [108] |
| Queensland Health | State health department | - Making tracks: Toward closing the gap in health outcomes for Indigenous Queenslanders by 2033 [109] |
| Royal Flying Doctor Service | Health charity specialist in medical retrieval and remote primary care | - Annual report 2011/12 - service provision at a local level, relevant to rural/remote [110]  - Looking Ahead: Responding to the health needs of country Australians in 2028/ the centenary year of the RFDS [111] |
| Rural and Regional Health Australia | www.ruralhealthaustralia.gov.au/internet/rha/publishing.nsf/Content/NSFRRH~HealthServices  (National Strategic framework for rural and remote health) | - National Strategic framework for rural and remote health [112] |
| Rural health west | Contains factsheets relating to, for example, distribution of particular health professionals in WA, useful for planning at a state level | - Specialist Services in Rural Western Australia: Gap and Equity [113] |
| Telethon Institute for Child Health Research | Research institute in Western Australia | - The Western Australian Aboriginal Child Health Survey: The Health of Aboriginal Children and Young People [114] |
| World Health Organisation | International standard setting | - Toolkit on monitoring health systems strengthening: Service Delivery [115] |

**References for tables in Appendix**

1. Achat HM, Thomas P, Close GR, Moerkerken LR, Harris MF. General health care service utilisation: where, when and by whom in a socioeconomically disadvantaged population. Australian Journal of Primary Health. 2010;16(2):132-40.

2. Adams E, Tongs J. Starting a perinatal and infant mental health service at Winnunga Nimmityjah. Australasian Psychiatry. 2011;19:S20-S2.

3. Allen O. Anthill and other injuries: a case for mobile allied health teams to remote Australia. Australian Journal of Rural Health. 1996;4(1):33-42.

4. Ansari Z, Laditka JN, Laditka SB. Access to health care and hospitalization for ambulatory care sensitive conditions. Med Care Res Rev. 2006;63(6):719-41. doi: 10.1177/1077558706293637.

5. Bailie RS, Togni SJ, Si D, Robinson G, d'Abbs PH. Preventive medical care in remote Aboriginal communities in the Northern Territory: a follow-up study of the impact of clinical guidelines, computerised recall and reminder systems, and audit and feedback. BMC health services research. 2003;3(1):15. doi: 10.1186/1472-6963-3-15.

6. Bailie RS, Si D, Dowden MC, Connors CM, O'Donoghue L, Liddle HE, et al. Delivery of child health services in Indigenous communities: implications for the federal government's emergency intervention in the Northern Territory. Med J Aust. 2008;188(10):615-8. Epub 2008/05/20. doi: 10.5694/j.1326-5377.2008.tb01806.x. PubMed PMID: 18484941.

7. Bar-Zeev SJ, Kruske SG, Barclay LM, Bar-Zeev NH, Carapetis JR, Kildea SV. Use of health services by remote dwelling Aboriginal infants in tropical northern Australia: a retrospective cohort study. BMC Pediatrics. 2012;12:19. doi: 10.1186/1471-2431-12-19.

8. Barclay L, Kruske S, Bar-Zeev S, Steenkamp M, Josif C, Narjic CW, et al. Improving Aboriginal maternal and infant health services in the 'Top End' of Australia; synthesis of the findings of a health services research program aimed at engaging stakeholders, developing research capacity and embedding change. BMC health services research. 2014;14(1):241. doi: 10.1186/1472-6963-14-241.

9. Barr M, Duncan J, Dally K. A systematic review of services to DHH children in rural and remote regions. The Journal of Deaf Studies and Deaf Education. 2018;23(2):118-30.

10. Breen C, Altman L, Ging J, Deverell M, Woolfenden S, Zurynski Y. Significant reductions in tertiary hospital encounters and less travel for families after implementation of Paediatric Care Coordination in Australia. BMC health services research. 2018;18(1):1-10.

11. Buckley D, Weisser S. Videoconferencing could reduce the number of mental health patients transferred from outlying facilities to a regional mental health unit. Australian and New Zealand Journal of Public Health. 2012;36(5):478-82. doi: 10.1111/j.1753-6405.2012.00915.x.

12. Chandradasa M, Basu S. Collaborative networking between regional child mental health, paediatric and educational services in Gippsland, Australia: An online survey. The Australian Journal of Rural Health. 2019.

13. Cord-Udy N. The Medical Specialist Outreach Assistance Programme in South Australia at 2 years. Australasian Psychiatry. 2004;12(2):161-5.

14. Couzos S. Practical measures that improve human rights - towards health equity for Aboriginal children. Health Promotion Journal of Australia : Official Journal of Australian Association of Health Promotion Professionals. 2004;15(3):186-92.

15. D'Aprano A, Silburn S, Johnston V, Bailie R, Mensah F, Oberklaid F, et al. Challenges in monitoring the development of young children in remote Aboriginal health services: clinical audit findings and recommendations for improving practice. Rural Remote Health. 2016;16(3):3852.

16. Dossetor DR, Nunn KP, Fairley M, Eggleton D. A child and adolescent psychiatric outreach service for rural New South Wales: a telemedicine pilot study. J Paediatr Child Health. 1999;35(6):525-9. doi: 10.1046/j.1440-1754.1999.00410.x.

17. Dossetor PJ, Martiniuk ALC, Fitzpatrick JP, Oscar J, Carter M, Watkins R, et al. Pediatric hospital admissions in Indigenous children: a population-based study in remote Australia. BMC Pediatrics. 2017;17(1):195. doi: 10.1186/s12887-017-0947-0.

18. Dossetor PJ, Thorburn K, Oscar J, Carter M, Fitzpatrick J, Bower C, et al. Review of Aboriginal child health services in remote Western Australia identifies challenges and informs solutions. BMC health services research. 2019;19(1):758. doi: 10.1186/s12913-019-4605-0.

19. Dossetor PJ, Fitzpatrick EFM, Glass K, Douglas K, Watkins R, Oscar J, et al. Emergency Department Presentations by Children in Remote Australia: A Population-based Study. Global Pediatric Health. 2021;8. doi: 10.1177/2333794X21991006.

20. Edmond KM, McAuley K, McAullay D, Matthews V, Strobel N, Marriott R, et al. Quality of social and emotional wellbeing services for families of young Indigenous children attending primary care centers; a cross sectional analysis. BMC health services research. 2018;18(1):1-11.

21. Edmond KM, Tung S, McAuley K, Strobel N, McAullay D. Improving developmental care in primary practice for disadvantaged children. Archives of Disease in Childhood. 2019;104(4):372-80.

22. Flabouris A, Hart GK, Nicholls A. Accessibility of the Australian population to an ICU, and of ICUs to each other. Critical care and resuscitation : journal of the Australasian Academy of Critical Care Medicine. 2012;14(3):177-84.

23. Francis A, Didsbury M, Lim WH, Kim S, White S, Craig JC, et al. The impact of socioeconomic status and geographic remoteness on access to pre-emptive kidney transplantation and transplant outcomes among children. Pediatric Nephrology. 2016;31(6):1011-9.

24. Garne DL, Perkins DA, Boreland FT, Lyle DM. Frequent users of the Royal Flying Doctor Service primary clinic and aeromedical services in remote New South Wales: a quality study. Med J Aust. 2009;191(11-12):602-4. doi: 10.5694/j.1326-5377.2009.tb03344.x.

25. Geelhoed GC, Geelhoed EA. Positive impact of increased number of emergency consultants. Archives of disease in childhood. 2008;93(1):62-4.

26. Gruen RL, Bailie RS, Wang Z, Heard S, O'Rourke IC. Specialist outreach to isolated and disadvantaged communities: a population-based study. Lancet. 2006;368(9530):130-8. doi: 10.1016/S0140-6736(06)68812-0.

27. Gunasekera H, Morris PS, Daniels J, Couzos S, Craig JC. Otitis media in Aboriginal children: the discordance between burden of illness and access to services in rural/remote and urban Australia. Journal of Paediatrics and Child Health. 2009;45(7-8):425-30.

28. Hayman N. Strategies to improve indigenous access for urban and regional populations to health services. Heart Lung Circulation. 2010;19(5-6):367-71. doi: 10.1016/j.hlc.2010.02.014.

29. Henderson R. Review of community paediatrics, the Central Australian Remote Health Service, Alice Springs. Prince Margaret Hospital for Children, Perth WA, 2008.

30. Humphreys JS. Key considerations in delivering appropriate and accessible health care for rural and remote populations: discussant overview. Australian Journal of Rural Health. 2009;17(1):34-8. doi: 10.1111/j.1440-1584.2008.01034.x.

31. Hussain R, Tait K. Parental perceptions of information needs and service provision for children with developmental disabilities in rural Australia. Disability and rehabilitation. 2015;37(18):1609-16.

32. Jeffery V, Ervin K. Responding to rural health needs through community participation: addressing the concerns of children and young adults. Australian Journal of Primary Health. 2011;17(2):125-30. doi: 10.1071/PY10050.

33. Johns S. Early childhood service development and intersectoral collaboration in rural Australia. Australian Journal of Primary Health. 2010;16(1):40-6.

34. Jones DM, McAllister L, Lyle DM. Rural and remote speech-language pathology service inequities: An Australian human rights dilemma. International journal of speech-language pathology. 2018;20(1):98-101.

35. Josif CM, Kruske S, Kildea SV, Barclay LM. The quality of health services provided to remote dwelling aboriginal infants in the top end of northern Australia following health system changes: a qualitative analysis. BMC Pediatrics. 2017;17(1):93. doi: 10.1186/s12887-017-0849-1.

36. Kang M, Robards F, Luscombe G, Sanci L, Usherwood T. The relationship between having a regular general practitioner (GP) and the experience of healthcare barriers: a cross-sectional study among young people in NSW, Australia, with oversampling from marginalised groups. BMC family practice. 2020;21(1):1-9.

37. Langbecker DH, Caffery L, Taylor M, Theodoros D, Smith AC. Impact of school-based allied health therapy via telehealth on children's speech and language, class participation and educational outcomes. Journal of Telemedicine and Telecare. 2019;25(9):559-65. doi: 10.1177/1357633X19875848.

38. Larson A, Bradley R. Aboriginal maternal and child project: strengths and needs analysis. Geraldton, WA: Combined Universities Centre for Rural Health and Aboriginal Health Council of Western Australia, 2010.

39. Lenthall S, Wakerman J, Opie T, Dunn S, Macleod M, Dollard M, et al. Nursing workforce in very remote Australia, characteristics and key issues. The Australian Journal of Rural Health. 2011;19(1):32-7.

40. Margolis SA. Is Fly in/Fly out (FIFO) a viable interim solution to address remote medical workforce shortages? Rural and Remote Health. 2012;12(4):1-6.

41. McCalman J, Tsey K, Clifford A, Earles W, Shakeshaft A, Bainbridge R. Applying what works: a systematic search of the transfer and implementation of promising Indigenous Australian health services and programs. BMC Public Health. 2012;12(1):600.

42. McKeown S. Evaluating aboriginal primary health care services using national key performance indicators: What has happened to the social theories of aboriginal health? Internal Medicine Journal. 2011;41:37.

43. Medlin L, editor Indigenous Respiratory Outreach Care (IROC): Enhancing Respiratory Health In Rural And Remote Aboriginal And Torres Strait Islander Communities. Respirology; 2014.

44. Mitchell AG, Belton S, Johnston V, Ralph AP. Transition to adult care for Aboriginal children with rheumatic fever: a review informed by a focussed ethnography in northern Australia. Australian Journal of Primary Health. 2018;24(1):9-13. doi: 10.1071/PY17069.

45. Mitchinson C, Strobel N, McAullay D, McAuley K, Bailie R, Edmond KM. Anemia in disadvantaged children aged under five years; quality of care in primary practice. BMC pediatrics. 2019;19(1):1-11.

46. Moffatt JJ, Eley DS. The reported benefits of telehealth for rural Australians. Australian Health Review. 2010;34(3):276-81. doi: 10.1071/AH09794.

47. Morgan K. QT babies: Neonatal care in the Queensland tropics. Journal of Paediatrics and Child Health. 2012.

48. Nancarrow SA, Roots A, Grace S, Saberi V. Models of care involving district hospitals: a rapid review to inform the Australian rural and remote context. Australian Health Review. 2015;39(5):494-507.

49. Nguyen KH, Smith AC, Armfield NR, Bensink M, Scuffham PA. Cost-Effectiveness Analysis of a Mobile Ear Screening and Surveillance Service versus an Outreach Screening, Surveillance and Surgical Service for Indigenous Children in Australia. PLoS One. 2015;10(9). doi: 10.1371/journal.pone.0138369.

50. Nguyen H, Zarnowiecki D, Segal L, Gent D, Silver B, Boffa J. Feasibility of implementing infant home visiting in a Central Australian Aboriginal community. Prevention Science. 2018;19(7):966-76.

51. O'Callaghan AM, McCallister L, Wilson L. Consumers' proposed solutions to barriers to access of rural and remote speech pathology services. Advances in Speech Language Pathology. 2005;7(2):58-64.

52. O'Kane A, Tsey K. Towards a needs based mental health resource allocation and service development in rural and remote Australia. Australas Psychiatry. 2004;12(4):390-5. doi: 10.1080/j.1440-1665.2004.02133.x.

53. Ou L, Chen J, Hillman K, Eastwood J. The comparison of health status and health services utilisation between Indigenous and non-Indigenous infants in Australia. Australian and New Zealand Journal of Public Health. 2010;34(1):50-6.

54. Peiris D, Wirtanen C, Hall J. Aeromedical evacuations from an east Arnhem Land community 2003-2005: the impact on a primary health care centre. Aust J Rural Health. 2006;14(6):270-4. doi: 10.1111/j.1440-1584.2006.00828.x.

55. Phillips JH, Wigger C, Beissbarth J, McCallum GB, Leach A, Morris PS. Can mobile phone multimedia messages and text messages improve clinic attendance for Aboriginal children with chronic otitis media? A randomised controlled trial. Journal of Paediatrics and Child Health. 2014;50(5):362-7.

56. Ruben AR, Fisher DA. The casemix system of hospital funding can further disadvantage Aboriginal children. Medical Journal of Australia. 1998;169(SUPPL.):S6-S10.

57. Schofield D, Fuller J, Wagner S, Friis L, Tyrell B. Multidisciplinary management of complex care. Australian Journal of Rural Health. 2009;17(1):45-8.

58. Seidler ZE, Rice SM, Dhillon HM, Cotton SM, Telford NR, McEachran J, et al. Patterns of youth mental health service use and discontinuation: Population data from Australia’s headspace model of care. Psychiatric services. 2020;71(11):1104-13.

59. Smith AC, Armfield NR, White MM, Williams ML, Koh T, Hurley T, et al. Clinical services and professional support: a review of mobile telepaediatric services in Queensland. Studies in Health Technology and Informatics. 2010;161:149-58.

60. Thomas SL, Wakerman J, Humphreys JS. What core primary health care services should be available to Australians living in rural and remote communities? BMC Family Practice. 2014;15:143. doi: 10.1186/1471-2296-15-143.

61. Veit FC, Sanci LA, Coffey CM, Young DY, Bowes G. Barriers to effective primary health care for adolescents. Medical Journal of Australia. 1996;165(3):131-3.

62. Verdon S, Wilson L, Smith-Tamaray M, McAllister L. An investigation of equity of rural speech-language pathology services for children: a geographic perspective. Int J Speech Lang Pathol. 2011;13(3):239-50. doi: 10.3109/17549507.2011.573865.

63. Wakerman J, Humphreys JS, Wells R, Kuipers P, Entwistle P, Jones J. Primary health care delivery models in rural and remote Australia: a systematic review. BMC health services research. 2008;8:276. doi: 10.1186/1472-6963-8-276. PubMed PMID: 19114003.

64. Wakerman J. Innovative rural and remote primary health care models: what do we know and what are the research priorities? Aust J Rural Health. 2009;17(1):21-6. doi: 10.1111/j.1440-1584.2008.01032.x.

65. Wakerman J, Humphreys JS, Wells R, Kuipers P, Jones JA, Entwistle P, et al. Features of effective primary health care models in rural and remote Australia: a case-study analysis. Medical Journal of Australia. 2009;191(2):88-91. doi: 10.5694/j.1326-5377.2009.tb02700.x.

66. Ward AM, de Klerk N, Pritchard D, Firth M, Holman CD. Correlations of siblings' and mothers' utilisation of primary and hospital health care: a record linkage study in Western Australia. Soc Sci Med. 2006;62(6):1341-8. doi: 10.1016/j.socscimed.2005.08.027.

67. Warwick S, Atkinson D, Kitaura T, LeLievre M, Marley JV. Young Aboriginal People's Perspective on Access to Health Care in Remote Australia: Hearing Their Voices. Prog Community Health Partnersh. 2019;13(2):171-81. doi: 10.1353/cpr.2019.0017.

68. Whelan S, Wright DJ. Health Services Use and Lifestyle Choices of Indigenous and non-Indigenous Australians. Social Science & Medicine. 2013.

69. Williams ML, Smith AC. Paediatric outreach services. Journal of Paediatrics and Child Health. 2004;40(9-10):501-3. doi: 10.1111/j.1440-1754.2004.00450.x.

70. Zwi K, Joshua P, Moran P, Casacelli M. Prioritising health services for high risk and disadvantaged children. Journal of Paediatrics and Child Health. 2011;47:23.

71. Crouchley K, Carlose N. Aboriginal Health Profile: Kimberley Health Region. WA: Department of Health, 2012.

72. WA Country Health Service. Kimberley Health Profile. Perth: WACHS, 2018.

73. DoH Epidemiology Branch. Child Population Profile, Health and Wellbeing Surveillance System, 2009 Kimberley health region. Perth: Department of Health, 2010.

74. Australian Indigenous HealthInfoNet. Overview of Aboriginal and Torres Strait Islander health status, 2020. 2021.

75. HealthInfoNet AI, Harford-Mills M, MacRae A, Drew N. Summary of Aboriginal and Torres Strait Islander health status 2018. 2019.

76. Australian Institute of Health and Welfare. Expenditure on Health for Aboriginal and Torres Strait Islander People 2008-09: An Analysis by Remoteness and Disease. AIHW, 2011.

77. Australian institute of Health and Welfare. Access to health services for Aboriginal and Torres Strait Islander people. 2011.

78. Australian institute of Health and Welfare. Aboriginal and Torres Strait Islander health services report, 2010-11: OATSIH services reporting - key results. Canberra: 2013.

79. Australian Institute of Health and Welfare. Healthy for Life-Aboriginal Community Controlled Health Services: Report Card. AIHW Canberra; 2013.

80. Australian institute of Health and Welfare. Aboriginal and Torres Strait Islander Health Performance Framework. Perth: 2013.

81. Australian Institute of Health and Welfare. Health workforce. Canberra: AIHW, 2020.

82. Australian Medical Association. Indigenous Health Report Card – "Ending the Cycle of Vulnerability: The Health of Indigenous Children". 2008.

83. Wakerman J, Humphreys J, Wells R, Kuipers P, Entwistle P, Jones J. A systematic review of primary health care delivery models in rural and remote Australia, 1993-2006. 2006.

84. McDonald L, Bailie R, Morris P, Rumbold A, Paterson B. Interventions to prevent growth faltering in remote Indigenous communities. Australian Primary Health Care Research Institute (APHCRI), The Australian National University; 2006.

85. Price E, Considine G. Kimberley Primary Health Care Sustainability Study 2008 - 2030. Kimberley Division of General Practice Ltd, 2008.

86. Sheperd C, Zubrick S. Human capabilities and child development: Some inferences using the 2008 NATSISS. Perspectives on the 2008 National Aboriginal and Torres Strait Islander Social Survey; ANU 2011.

87. Shaw G. An ethnography of changes in child rearing over time in the Ngaanyatjarra Lands: Implications for policy development on health in early childhood. CAEPR Seminar Series Canberra2013.

88. COAG Reform Council. Indigenous reform 2011-12: comparing performance across Australia. 2013.

89. Humphreys J, Wakerman J. Primary health care in rural and remote Australia: achieving equity of access and outcomes through national reform: A discussion paper. Canberra: National Health and Hospitals Reform Commission, 2008.

90. Allen, Clarke. Evaluation of the child health check initiative and the expanding health service delivery initiative: summary report. Department of Health and Ageing Canberra; 2011.

91. Standing Committee on Family and Community Affairs. Standing Committee on Family and Community Affairs. Health is life: Inquiry into indigenous health. Canberra: 2000.

92. Standing community on Health and Ageing. The Blame Game: Report on the inquiry into health funding. Canberra: Senate, 2006.

93. The Senate Select Committee on Regional and Remote Communities. Indigenous Australians, Incarceration and the Criminal Justice System. Canberra: Senate, 2010.

94. Health Workforce Australia. National Rural and Remote Workforce Innovation and Reform Strategy. Adelaide: HWA, 2013.

95. Health Workforce Australia. Health Workforce 2025 – Doctors, Nurses and Midwives – Volume 2. Adelaide: HWA, 2012.

96. Health Workforce Australia. Growing Our Future: the Aboriginal and Torres Strait Islander Health Worker Project Final Report. Adelaide: HWA, 2011.

97. Atkinson D, Bridge C, Gray D. Regional Aboriginal Health Plan: Aboriginal Health in the Kimberley: current circumstances and future directions. 2000.

98. Lewis J, editor Kimberley aboriginal primary health plan 2012-2015. Kimberley Aboriginal Health Planning Forum Broome; 2013.

99. Vos T, Carter R, Barendregt J, Mihalopoulos C, Veerman L, Magnus A, et al. Assessing cost-effectiveness in prevention: ACE–prevention September 2010 final report: University of Queensland; 2010.

100. Freemantle C, Officer K, Mcaullay D, Anderson I. Australian Indigenous Health-Within an International Context. Darwin: Cooperative Research Centre for Aboriginal Health, 2007.

101. Sanchez L, Carney S, Estermann A, Sparrow K, Turner D. An evaluation of the benefits of swimming pools for the hearing and ear health status of young Indigenous Australians: A whole-of-population study across multiple remote Indigenous communities. Adelaide: Flinders University. 2012.

102. Lowitja Institute. Close the gap campaign report 2021. 2021.

103. Menzies School of Health Research. Effective Integration of Services for Children

and Families: Making it Happen. Darwin: 2011.

104. Thomas S. Kids Pitstop: one stop community health shop for Indigenous families in the West Pilbara. Rural Health: the place to be; 17-20 May; Cairns 2009.

105. Grant S. Child Malnutrition / Failure to Thrive Project 2008: Final Report. Darwin, NT.: Department of Health and Families, Northern Territory Government, 2008.

106. Office of Aboriginal and Torres Strait Islander Health. Improving Health in Aboriginal and Torres Strait Islander Mothers, Babies and Young Children - A Literature Review. 2005.

107. Griew R, Tilton E, Cox N, Thomas D. The link between primary health care and health outcomes for Aboriginal and Torres Strait Islander Australians. Report for the Office of Aboriginal and Torres Strait Islander Health Canberra: DoHA. 2008.

108. Office for Aboriginal Torres Strait Islander Health. Better health care: studies in the successful delivery of primary health care services for Aboriginal and Torres Strait Islander Australians. Canberra: Commonwealth Department of Health and Ageing, 2001.

109. Queensland Health. Making Tracks toward closing the gap in health outcomes for Indigenous Queenslanders by 2033 Implementation Plan 2009-10 to 2011-12. Brisbane: Department of Health, 2010.

110. Royal Flying Doctor Service. Annual Report 2011-12. 2012.

111. Gardiner F, Gale L, Ransom A, Laverty M. Looking Ahead: responding to the health needs of country Australians in 2028-the centenary year of the RFDS. Canberra, Australia: The Royal Flying Doctor Service. 2018.

112. Department of Health and Ageing. National Strategic Framework for Rural and Remote Health. 2011.

113. Rural Health West. Specialist Services in Rural Western Australia: Gap and Equity Perth: RHW, 2013.

114. Zubrick SR, Lawrence DM, Silburn SR, Blair E, Milroy H, Wilkes T, et al. The Western Australian Aboriginal Child Health Survey: The Health of Aboriginal Children and Young People. Perth: Telethon Institute for Child Health Research, 2004.

115. World Health Organization. Toolkit on monitoring health systems strengthening: Service Delivery. 2008.
