# Supplementary material for: Health services for aboriginal and Torres Strait Islander children in remote Australia: A scoping review
Source: PLOS Glob Public Health. 2023 Feb 13;3(2):e0001140. doi: 10.1371/journal.pgph.0001140 (PMC10022200; doi:10.1371/journal.pgph.0001140)
Supplement: Attachment — Submitted filename: Response to reviewers.docx [file pgph.0001140.s003.docx]

**RE: PLOS Global Public Health: Revision (PGPH-D-22-01341) - [EMID:43b602e6274096fa]**

**Dec 14 2022**

Health services for Aboriginal and Torres Strait Islander children in remote Australia: a scoping review

Response to Editor and Reviewers

Our responses in RED

# Editor

1. ***Please indicate the full and correct funding information for your study and confirm the order in which funding contributions should appear in the online submission.***

Updated and numbered in manuscript before reference list.

2. ***We have noticed that you have uploaded Supporting Information files, but you have not included a list of legends. Please add a full list of legends for your Supporting Information files after the references list.***

A legend has been added after references in the manuscript.

Please review your reference list to ensure that it is complete and correct. If you have cited papers that have been retracted, please include the rationale for doing so in the manuscript text, or remove these references and replace them with relevant current references. Any changes to the reference list should be mentioned in the rebuttal letter that accompanies your revised manuscript. If you need to cite a retracted article, indicate the article’s retracted status in the References list and also include a citation and full reference for the retraction notice.

Checked with thanks.

# Reviewer 1

Thank you for your thoughtful and lengthy review of our submission. You helpfully contextualised our work and did not suggest any specific revisions that we should address here.

# Reviewer 2

***The authors indicate that 'review by an ethical board and consent was not appropriate'. I understand the premise behind this since this is a scoping review. I would have expected to see a "exempt" determination coming from the Ethical Review Board of the primary institution (University of Sydney?)- particularly since work seems to have originated from a PhD thesis. I would like the journal to determine if this statement is adequate.***

Obtaining research ethics approval or exemption for literature reviews of the existing peer-reviewed, published literature is not typical or required practice currently. The authors are aware of the global academic debate about whether formal ethical review in advance of conducting literature reviews might be required or could be useful in the future. Our impression is that this is not standard practice, nor is it likely to become so. Ethical review norms are a dynamic area, and the authors will take advice from the Journal.

1) ***In the Abstract - "Gold standard...". Perhaps using "Best practices" would convey the message better.***

Edited to say ‘best practices’ now, thank you.

2) ***Line 5-8. The sentence is incomplete. I recommend dropping "which" or altering the sentence.***

Thank you, the sentence has been revised for clarity.

3) ***Line 12-13. "The Indigenous..." sentence is confusing. Please review and rewrite the sentence.***

Re-written, thank you for highlighting.

4) ***Line 99 and 100 - why were articles dealing with substance abuse and misuse and health policy articles excluded. Later in the manuscript I see references to policy articles.***

Our scoping review aimed to capture health services for Indigenous children in remote areas of Australia. It included services which treated substance misuse however.

5) ***Line 229, 230. I am not sure how these "other measures" may not be relevant. I acknowledge the importance of primary care services, but the other measures are also important to consider.***

This has been re-written – the original text didn’t convey our meaning: measures that usefully describe how rurality impacts health care access become less useful in remote settings.

6) ***Line 237,238, 239. Comparing across jurisdictions is normal practice in many health services research- and should not be because each jurisdiction manages their own budget. You also do a comparison in line 324, 325. I would recommend eliminating this sentence.***

Thank you for this comment. We have edited this section of the manuscript accordingly.

7) ***Line 263, 264. I am not sure what NT or WA are. I would recommend using the full form, not abbreviations, when used for the first time. This is partly because this journal has a global audience.***

Abbreviations are now written in full in the first instance.

8) ***Line 362, 363. It would get a sense of what this 5.6 FTE represents. Is it a ratio? ( "per 100 FTEs") or if it is an absolute number then please indicate what the total FTE is.***

Rewritten with ‘denominator’ provided, thank you for noting.

9) ***Line 566. Again, I would recommend "Best practices" rather than "Gold Standard"***

Changed with thanks.
